# DRG coding practice: a nationwide hospital survey in Thailand

**DOI:** 10.1186/1472-6963-11-290

**Published:** 2011-10-31

**Authors:** Krit Pongpirul, Damian G Walker, Hafizur Rahman, Courtland Robinson

**Affiliations:** 1 Department of Preventive and Social Medicine, Faculty of Medicine, Chulalongkorn University, Bangkok, Thailand; 2 Department of International Health, Johns Hopkins Bloomberg School of Public Health, Baltimore, USA

## Abstract

**Background:**

Diagnosis Related Group (DRG) payment is preferred by healthcare reform in various countries but its implementation in resource-limited countries has not been fully explored.

**Objectives:**

This study was aimed (1) to compare the characteristics of hospitals in Thailand that were audited with those that were not and (2) to develop a simplified scale to measure hospital coding practice.

**Methods:**

A questionnaire survey was conducted of 920 hospitals in the Summary and Coding Audit Database (SCAD hospitals, all of which were audited in 2008 because of suspicious reports of possible DRG miscoding); the questionnaire also included 390 non-SCAD hospitals. The questionnaire asked about general demographics of the hospitals, hospital coding structure and process, and also included a set of 63 opinion-oriented items on the current hospital coding practice. Descriptive statistics and exploratory factor analysis (EFA) were used for data analysis.

**Results:**

SCAD and Non-SCAD hospitals were different in many aspects, especially the number of medical statisticians, experience of medical statisticians and physicians, as well as number of certified coders. Factor analysis revealed a simplified 3-factor, 20-item model to assess hospital coding practice and classify hospital intention.

**Conclusion:**

Hospital providers should not be assumed capable of producing high quality DRG codes, especially in resource-limited settings.

## Background

Since 2001, the Universal Coverage (UC) scheme has provided health benefits to approximately three quarters of Thailand's citizens. The scheme is financed from general taxation and administered by the National Health Security Office (NHSO), under the supervision of the Public Health Minister. Hospital providers are mainly from the public sector and are paid for outpatient and preventive services based on prospective capitation whereas the Diagnosis Related Group (DRG)-based retrospective payment was used to compensate for the cost of inpatient care.

The DRG system can work well if the diagnosis and procedure codes could reflect both the patient's clinical condition and the actual cost of care incurred by hospital providers. In addition, coding practice in hospitals has to be reliable and capable of producing consistent and reliable coding quality. In an ideal setting, physicians would carefully review relevant clinical information to prepare a discharge summary, which would then be used by certified coders to produce appropriate diagnosis and procedure codes to be submitted for reimbursement. However, such an ideal condition is unlikely, at least in resource-limited settings like Thailand.

In a related qualitative study, we detailed the variation of coding practice in 10 hospitals and then defined the concept of Hospital Coding Practice as comprising elements of both structure and process [1]. In terms of structure, we identified at least eight health care professional disciplines (Medical Statisticians, Nurse, Physician, Public Health Staff/Paramedics, Medical Record Staff, Information Technology Staff, Finance/Accounting Staff, and others) as well as IT infrastructure specifically relevant to coding practice. We also described seven major steps of the coding process (Discharge Summarization, Completeness Checking, Diagnosis and Procedure Coding, Code Checking, Relative Weight Challenging, Coding Report, and Internal Summary and Coding Audit). The findings demonstrated that coding practice is not a simple two-step activity as had been assumed before by payers, but rather a multi-step process that involves many overlapping responsibilities across health care professional disciplines, especially in a resource-limited setting [1].

Although DRG has been applied in many countries and has been improved over the decades, it still is an imperfect system and a number of concerns have therefore been raised. Discrepancies between the submitted codes and the information in medical records revealed during the coding audit--especially when the submitted codes can result in larger reimbursements than would be consistent with the actual condition--have triggered a number of concerns about quality of medical records, cooperation of physicians, knowledge and skill of coders, as well as hospital intentions to "game the system" (also known as "DRG Creep") [2]. While most of these issues can be objectively verified, the last concern seems to be both the most important and the most difficult to measure.

Hospitals in Thailand are required to check for the accuracy and completeness of the data using the National Health Security Office (NHSO) standard guidelines [3]; however, errors have been frequently reported. Each hospital is therefore required to check the data before submission, and penalties--financial and non-financial--are imposed if errors are found. In 2008, the Bureau of Claims and Medical Audit (BCMA) conducted the Summary and Coding Audit on 57,828 medical records of 931 hospitals in 75 provinces (SCAD 2008). Hospitals were selected based on pre-specified criteria as presented in Table 1[4]. These 'SCAD hospitals' were then regarded negatively as they were suspected of either having poor data quality or trying to manipulate the system.

**Table 1:** Inclusion criteria for SCAD 2008

Hospitals that:
Are in the top 100 for reimbursement amount, after sorting for Adjusted Relative Weight (RW)

Have large amount of 'abnormal' values defined as:

- Procedure does not match hospital capacity

- Diagnosis and procedure codes are not correct

- Admission of ambulatory cases

- Larger among of reimbursement than other hospitals with similar size

- 'High-cost DRG (RW > 4)



**Medical record that has:**

- RW >= 8 but Length of Stay (LOS) < 3

- RW < 5 but LOS > 10 or > 2 times the average LOS of the same DRG

- Tracheostomy procedure code but LOS < 3 and discharge status = cure

- Cardiovascular surgery procedure code that is not relevant to the hospital capacity

- Brain surgery procedure code but LOS < 3 and discharge status = improved

- Kidney dialysis procedure code but LOS < 5

- Large amount of data in some DRG

- Adjusted RW does not concur with cost

- Other unusual conditions

Source: Pongpirul and Wongkanaratanakul [4]

Note: RW = Relative Weight, LOS = Length of Stay (day)

Hospital coding practice can be affected by some factors beyond a hospital's control [1]. Simply because the hospitals were chosen to be audited based on the above inclusion criteria does not necessarily mean they intended to cheat or were incapable of producing quality coding. Understanding what might distinguish SCAD hospitals from the non-SCAD counterparts was an appropriate and feasible next step. This nationwide hospital survey has two main objectives: (1) to describe and compare the characteristics of both groups of hospitals and (2) to develop a simplified scale to measure hospital coding practice.

## Methods

### Questionnaire

Based on the case study findings [1], a questionnaire was developed to comprise three sections. Section 1 asked about general demographics of the respondents and of the hospitals. Section 2 aimed to explore the hospital coding structure; various types of resources in the hospital were asked. Section 3.1 explored how, for each step of the hospital coding process, primary and secondary responsibility was assigned to, or assumed by, a particular health care professional discipline. Face validity of the questionnaire was assessed by presenting the findings to data-coding experts. The general demographic information was verified with national hospital registry to ensure content validity. To ensure comprehensibility and feasibility, a pilot test was conducted among 3-5 graduate students with relevant professional background as well as among a few public and private hospitals; feedback was then used for revision. Forward and backward translation was done to ensure its conceptual and linguistic equivalence.

Section 3.2 contained a set of opinion-oriented items to reflect on the current hospital coding practice. A pool of 63 items were identified from the interviews with the 10 hospitals in the case study [1]. They were categorized into group A to K, according to their relevance to each step of the hospital coding process (A1-A19: Non-specific; B1-B8: Discharge Summarization; C1-C2: Completeness Checking; DE1-DE10: Diagnosis and Procedure Coding; F1-F6: Code Checking; G1-G5: Relative Weight Challenging; H1-H3: Coding Report; I1-I3: Medical Record Audit; J1-J3: Internal Summary Audit; K1-K3: Internal Coding Audit). These items were presented as declarative sentences, followed by response options that indicate varying degree of agreement with the statement. A five-point Likert scale format (-2 = Strongly Disagree; -1 = Disagree; 0 = Neutral; +1 = Agree; +2 = Strongly Agree) was considered the most appropriate, based on the feedback from our pilot test, for the respondents' ability to discriminate meaningfully [5].

### Site & Study Population

**Figure 1:**
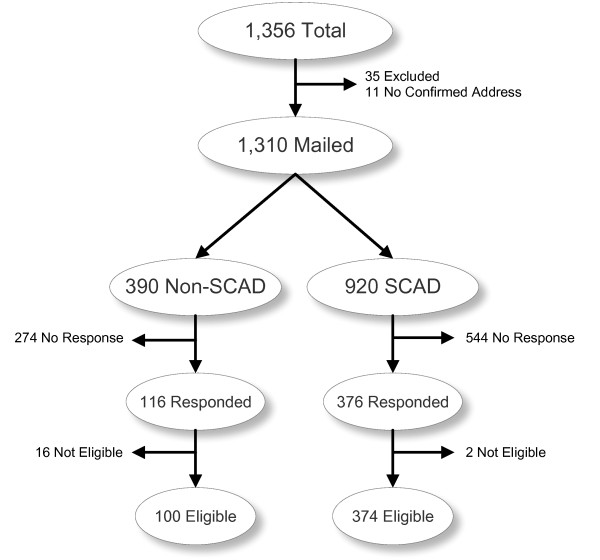
**Survey strategy**.

The study population of interest initially included all 1,356 hospitals in Thailand, of which 931 hospitals participated in the UC scheme and their audit results were contained in the SCAD 2008. Thirty-five hospitals that provided services to highly specific population groups (such as drug addicts, prisoners, special psychiatric patients, organizational employees) or had special kind of financial support, and 11 hospitals without verified postal address were excluded. After exclusion, there were 1,310 target hospitals, comprising 920 SCAD and 390 non-SCAD hospitals (Figure 1). The respondents targeted for the study were hospital staff who were responsible for the diagnosis and procedure coding process, as identified by the hospital director.

### Data Collection

The questionnaire, a cover letter, and a prepaid return envelope were mailed to the target hospitals. Hospitals were asked to return the questionnaire within four weeks, after which two follow-up phone calls were done. Non-respondents were defined as those who did not return the questionnaire within two weeks after the second follow-up call. This study was determined by the Johns Hopkins Bloomberg School of Public Health Institutional Review Board as not human subjects research (IRB# 00002096).

### Data Analysis

For responses to the Section 1 (General Demographics), Section 2 (Hospital Coding Structure), and Section 3.1 (Hospital Coding Process), Pearson's chi-square and Student's t-test were used for analyzing categorical and continuous variables, respectively. Exploratory factor analysis (EFA) was used to analyze the responses in Section 3.2 (Hospital Coding Practice Scale). EFA is a technique used to explain covariance among observed random variables in terms of fewer unobserved random variables named factors. It helps to generate a hypothesis in such a way that the investigation of the relationships between manifest variables and factors is not based on any prior assumptions about which manifest variables are related to which factors [6].

The factor analysis was done to identify optimal number of factors, determined by Kaiser-Guttman Criterion (Number of eigenvalues > 1) [7], scree test [8], and parallel analysis [9]. Items with high uniqueness, defined as larger than 0.70, were removed whereas the remaining items were retained within the factors that showed high factor loadings. According to the 'rule of thumb' in confirmatory factor analysis, loadings should be at least 0.70 because it corresponds to about half of the variance of the variable being explained by the factor of interest. However, some researchers suggest lower levels of cut-points such as 0.60 [10] or as low as 0.40 [11] for EFA. We also performed initial reliability test and item-based statistics in conjunction with EFA [5]. Stata/SE Version 10 (Stata Corp.) was used for all statistical calculations.

## Results

### Response

The overall response rate was 37.56% with a well-balanced geographical distribution. Of those who responded, 18 hospitals were excluded because they were no longer in operation. The sample therefore consisted of 374 SCAD and 100 non-SCAD hospitals. The SCAD hospitals were significantly more likely to respond than Non-SCAD hospitals (40.87% vs 29.74%; OR 1.63; 95% CI: 1.26-2.12; p = 0.0001). Public hospitals were significantly more likely to respond to our survey than private hospitals (p < 0.001). The geographical distributions were similar between the responders and non-responders (p = 0.663). Larger hospitals were significantly more likely to respond to the survey than smaller ones (p < 0.001).

### Characteristics of Hospital Survey Responders

The majority of the contact persons was female (80.08%) with an average age of 37 years and had worked in their hospital for at least 12 years on average (Table 1). Almost half of them (47.97%) were medical statisticians and 31.71% were nurses. Each of the surveyed hospitals was responsible for an average UC population of 60,000. Universal Coverage, moreover, was the major source of hospital revenue for most responders. Under the UC scheme, hospitals have had to set up an adequate number of Primary Care Units (PCU) to provide care for remote populations. In this survey, the mean number of PCUs was 9.20 per hospital. On average, approximately 75% of the beds were occupied. The average length of stay (LOS) was 5.95 days. The mean Relative Weight (RW)--a standard value assigned to each DRG to reflect the cost of its care--and Adjusted Relative Weight (Adjusted RW) were 0.91 and 0.82, respectively. On an average day, the surveyed hospitals took care of 75.50 inpatient and 511.23 outpatient cases. One quarter of the hospitals received full accreditation by the Healthcare Accreditation Institute (similar to the US's Joint Commission on Accreditation of Healthcare Organizations). SCAD hospitals were more likely to be public and smaller than non-SCAD hospitals (p < 0.001). The majority of SCAD hospitals were in Northeast and Central region whereas non-SCAD hospitals were from Bangkok and Central region.

### Hospital Coding Structure

The number of computers used specifically for coding purposes in each hospital ranged from 1 to 200 (Mean 7; SD 14.56; N = 376), regardless of SCAD status. We found a significant variation in the types of software that hospitals used. These software programs offered different capability to assist in the coding process. Even the most popular software (HOSxP) was used in only 45% of the hospitals sampled. Majority of SCAD hospitals used HOSxP whereas most of the Non-SCAD hospitals preferred either less common commercial or proprietary software.

We reported at least eight health care professional disciplines (Medical Statisticians, Nurse, Physician, Public Health Staff/Paramedics, Medical Record Staff, Information Technology Staff, Finance/Accounting Staff, and others) were involved in the hospital coding practice [1]. The number of medical statisticians as well as experience of medical statisticians and physicians were statistically significantly different between SCAD and non-SCAD hospitals (p = 0.0256) (Table 2).

**Table 2:** Human resource for hospital coding practice in SCAD and Non-SCAD hospitals

	Overall	SCAD	Non-SCAD	p-value
N	465	370	95	

Total # Staff Involved	7.52 ± 14.32	7.49 ± 14.18	7.64 ± 14.91	p = 0.9287

Medical Statisticians (2&4 yr)				

# Staff	1.79 ± 1.41	1.72 ± 1.31	2.22 ± 1.86	p = 0.0256

# Training (times)	3.24 ± 3.34	3.27 ± 3.44	2.83 ± 1.18	p = 0.8623

Experience (years)	10.74 ± 5.49	10.63 ± 5.58	12.17 ± 4.85	p = 0.6465

Medical Statisticians (2 yr)				

# Staff	1.25 ± 1.11	1.21 ± 1.00	1.46 ± 1.60	p = 0.1658

# Training (times)	3.62 ± 3.30	3.60 ± 3.35	3.75 ± 2.92	p = 0.8525

Experience (years)	8.11 ± 6.01	7.89 ± 5.92	9.69 ± 6.55	p = 0.1372

Medical Statisticians (4 yr)				

# Staff	0.54 ± 1.03	0.50 ± 0.92	0.76 ± 1.54	p = 0.1189

# Training (times)	4.44 ± 5.31	4.58 ± 5.49	2.83 ± 1.47	p = 0.4430

Experience (years)	11.67 ± 6.19	12.15 ± 5.95	7.70 ± 7.05	p = 0.0310

Nurse				

# Staff	5.05 ± 9.58	5.41 ± 10.23	3.67 ± 6.30	p = 0.2324

# Training (times)	2.70 ± 2.25	2.74 ± 2.35	2.63 ± 1.79	p = 0.7856

Experience (years)	4.80 ± 3.27	4.75 ± 3.36	5.05 ± 3.02	p = 0.5898

Physician				

# Staff	4.06 ± 6.74	3.74 ± 5.08	6.67 ± 14.08	p = 0.0838

# Training (times)	1.96 ± 1.35	1.93 ± 1.34	2.33 ± 1.63	p = 0.4890

Experience (years)	3.10 ± 2.46	2.89 ± 2.24	5.36 ± 3.71	p = 0.0109

Public Health Staff				

# Staff	2.06 ± 2.24	2.10 ± 2.34	1.00 ± 0.00	p = 0.5159

# Training (times)	2.11 ± 2.23	2.00 ± 2.32	3.00 ± 1.41	p = 0.5643

Experience (years)	3.05 ± 2.20	3.22 ± 2.31	2.04 ± 1.47	p = 0.7523

Medical Record Staff				

# Staff	2.93 ± 3.11	2.96 ± 2.84	2.89 ± 3.51	p = 0.9220

# Training (times)	2.38 ± 2.03	2.39 ± 2.59	2.42 ± 1.41	p = 0.9618

Experience (years)	6.86 ± 6.96	7.30 ± 7.25	6.61 ± 6.85	p = 0.6357

IT Staff				

# Staff	2.22 ± 3.87	2.42 ± 4.28	1.20 ± 0.45	p = 0.5340

# Training (times)	1.64 ± 0.92	1.78 ± 0.97	1.00 ± 0.00	n/a

Experience (years)	1.37 ± 0.71	1.67 ± 0.52	5.00 ± 0.00	n/a

Finance/Accounting Staff				

# Staff	2.10 ± 2.23	1.82 ± 2.21	3.25 ± 2.22	p = 0.2609

# Training (times)	2.20 ± 1.10	2.20 ± 1.10	n/a	n/a

Experience (years)	3.08 ± 1.30	3.08 ± 0.27	n/a	n/a

Others				

# Staff	4.39 ± 9.35	3.59 ± 7.29	6.34 ± 12.91	p = 0.1947

# Training (times)	1.92 ± 1.92	1.88 ± 1.81	2.13 ± 2.30	p = 0.6820

Experience (years)	5.26 ± 6.66	4.33 ± 4.32	7.50 ± 10.11	p = 0.0488

**Figure 2:**
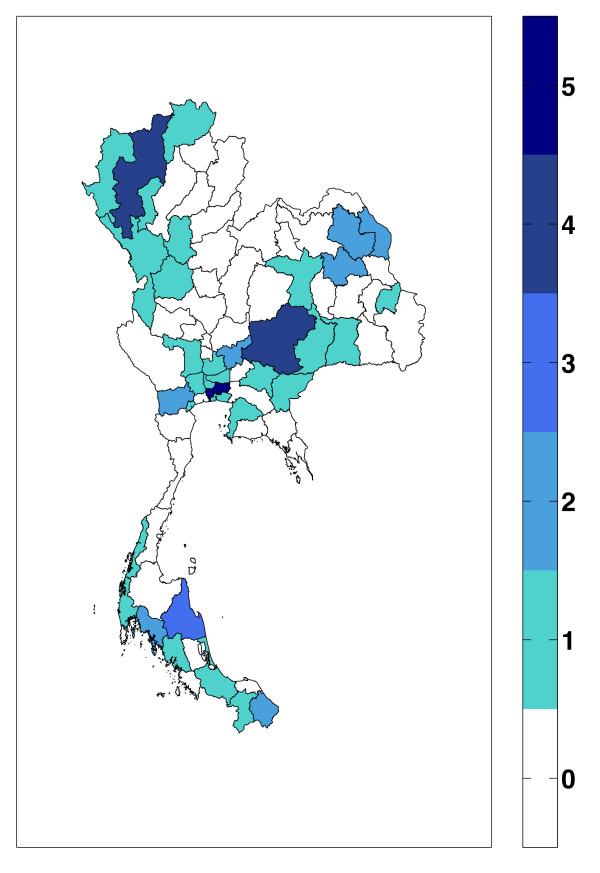
**Distribution of medical statisticians with 2- or 4-year degree program**.

Only 55 out of 492 hospitals (11.18%) reported that they had at least one formally trained medical statistician (Figure 2). There were at least 572 medical statisticians who were formally trained and received the 2-year certificate program from Kanchanabhisek Institute of Medical and Public Health Technology (KMPHT). Approximately 30% of them continued their study to finish the 4-year Bachelor's of Science Program in Medical Records from the Department of Social Science, Faculty of Social Sciences and Humanities, Mahidol University.

Regardless of formal training, some of the experienced hospital staff could take an examination to be certified as intermediate and advance coders, who could also be invited to work as external auditors upon request. SCAD hospitals were more likely to have fewer certified coders than non-SCAD hospitals (Table 3).

**Table 3:** Certified intermediate and advance coders

	Overall	SCAD	Non-SCAD	p-value
N	274	237	37	

Intermediate Coder	0.74 ± 1.13	0.63 ± 1.04	1.49 ± 1.35	p < 0.001

Advance Coder	0.04 ± 0.29	0.04 ± 0.31	0.03 ± 0.16	p = 0.8337

### Hospital Coding Process

**Figure 3:**
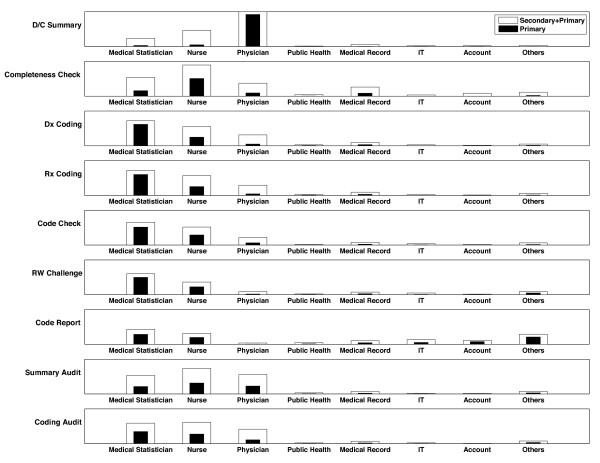
**Primary and secondary responsible staff in each step of the hospital coding process**.

Hospital coding process involved different health care professional disciplines in each of the steps. Figure 3 depicts the proportional representation of both primary and secondary responsible staff for all steps of the hospital coding process. The distribution of primary and secondary responsible staff between SCAD and non-SCAD hospitals are not different (results not shown).

### Hospital Coding Practice Scale

Based on the Kaiser-Guttman criterion, scree test, and parallel analysis, our data revealed that the hospital coding practice should comprise 2-4, 10, and 15 factors, respectively. Although parallel analysis has been considered the most accurate as compared to the other two criteria, it suggested a 15-factor model which we thought was not simple enough for our exploration of hospital coding practice.

We then explored the Kaiser-Guttman criterion by re-running the factor analysis with 10 factors specified. The factors were rotated to spread variability more evenly among factors so that all solutions are relatively the same. We used a cut point of 0.70 to drop items with high uniqueness, grouped the retained items into factors, and name the factors based on the member items. We found that 10-factor model was still not clear as some items have similar loadings across 2-3 factors. For example, item A10 (There is a physician responsible for coding practice) had 0.4275 and 0.4613 loadings for factor 2 and 5, respectively.

We therefore tried to follow the scree test approach and re-run the analysis with 4 factors. Orthogonal (varimax) and oblique (promax) rotation gave similar results but we decided to proceed with the latter because of the potential non-independent nature of the factors. After rotation and deletion of items with high uniqueness, we found that Factor 4 did not contain any items. Hence, the findings suggested that 3 factors might be the best solution for our purpose (Table 4).

**Table 4:** Grouping and naming 3 factors

Loadings	FACTOR 1 - DATA QUALITY
0.7810	Summary Audit is always conducted

0.7793	Results of Summary Audit are informed and used to improve discharge summary

0.7246	Results of Medical Record Audit are used for improvement

0.6939	There is a committee responsible for Summary Audit

0.6931	Coding Audit is always conducted

0.6849	Results of Medical Record Audit are publicly announced

0.6719	Medical Record Audit is always conducted

0.6591	Results of Coding Audit are informed and used to improve coding

0.5940	There is a committee responsible for Coding Audit

0.5789	There is a committee responsible for Medical Record Audit

	**FACTOR 2 - CODING PRACTICE**

0.6268	There is a policy to ensure that physicians do the codings themselves

0.6011	Physician has to provide diagnosis and procedure codes in the discharge summary

0.5544	The hospital develops a computer software to check the codes given by coder

0.5527	There is a physician responsible for coding practice

0.4844	There is an incentive/punishment mechanism to ensure timeliness of the discharge summary completion

0.4413	The hospital provides clear career pathway for medical statistician

0.4094	There is a staff responsible for using DRG Seeker software

	**FACTOR 3 - REIMBURSEMENT**

0.7626	Various combinations of codes are entered into DRG Seeker software to see the change in RW/Adjusted RW

0.7310	Principal and secondary diagnoses may be swapped in order to see the change in Adjusted RW

0.5849	DRG Seeker software is used for every inpatient

	**Uniqueness > 0.70**

0.5818	Health insurance status will be checked before coder can code

0.4935	Checking health insurance status is an essential step of coding practice

0.4876	There have been attempts to find diagnosis and procedure codes that give as high RW as possible

0.3768	A criteria is used to select some discharge summary for code checking

0.3440	Coder always needs to see laboratory results

Factor 1 was named "Data Quality" as hospitals who score high for this factor seemed to pay more attention to the quality of medical record, discharge summary, as well as diagnosis and procedure codes. One could see that hospitals who score high for the second factor paid attention to various aspects of coding practice. Factor 2 was therefore called "Coding Practice". Factor 3 contained three items, all of which suggested relevance to a hospital's interest in reimbursement rather than data quality or coding practice. Hence, we named it "Reimbursement". We also revisited the other items that were dropped because of high uniqueness but also had considerable loadings to Factor 3. Interestingly, they were also suggestive of hospital's reimbursement interest but with the other step of hospital coding process. Table 5 presents the three factors as three different profiles.

**Table 5:** Hospital intention profiles based on the 3-factor model

Profile	Data Quality	Coding Practice	Reimbursement	Description*
1	X			Hospital appoints committees to be responsible for the quality of medical record, discharge summary, as well as diagnosis and procedure codes. The audits are regularly conducted and the findings are used as feedback to improve the quality of medical records, discharge summary, as well as diagnosis and procedure codes.

2		X		Hospital sets policy and provides essential inputs to ensure good diagnosis and procedure codes. Physicians are considered as the key profession and therefore expected to take responsibility at various levels. Hospital may appoint a physician to oversee the whole hospital coding practice. Physicians at operational level may be required to provide appropriate diagnosis and procedure codes, in addition to their mandated discharge summary task. Medical statisticians are important primarily for checking the codes provided by the physicians and secondarily overseeing the IT aspect of the coding process.

3			X	Hospital pays most of its attention on the diagnosis and procedure codes given. The codes are strategically analyzed to see how much relative weight would change across various sets of codes. Selectivity is more obvious when health insurance status of the patient has to be checked before the coding is done.

4	X	X		Hospital not only tries to improve coding practice, but also attempts to monitor the quality of medical record, discharge summary, as well as diagnosis and procedure codes.

5		X	X	Hospital tries to improve coding practice, but mainly to increase relative weight and therefore reimbursement.

6	X		X	Hospital concerns about both data quality and it effects on reimbursement; however, no explicit evidence of coding practice improvement can be found.

7	X	X	X	Hospital shows evidence of improving the coding practice, monitoring the quality of medical record, discharge summary, and diagnosis and procedure codes, as well as checking how the codes affect final reimbursement amount.

8				Hospital is in status quo with no interest in monitoring data quality, improving coding practice, or how much reimbursement would be affected.

Note: Each of the profile is described based on the set of items in all factors included in the profile.

## Discussion

As a preferred method for provider payment in both developed and developing countries [12], DRG assumes that hospitals are well equipped with physicians and certified coders and therefore able to submit diagnosis and procedure codes with high quality. Literature on DRG implementation has been mostly from countries with abundant resource or mainly about its macro-level effects whereas study on how DRG codes are actually produced by hospitals is lacking.

DRG creep has been a major concern in resource-rich setting, in which hospital is suspected of being profit maximizer. As this kind of organizational intention is difficult to assess directly, it is not surprising to see mixed findings on the extent of DRG creep based on surrogate outcome measures [13-18]. While some studies investigated organizational behavior and reported potential influence of hospital management and payer on coding process [19,20], other studies tried to demonstrate potential upcoding of some specific diagnoses such as pneumonia and heart failure [21,22].

Poor coding quality is not only because of DRG creep, but also sicker patients, improvements in coding, and changes instituted by the payer [13]. We added that variation in hospital coding practices in an under-resourced health system is another major determinant of DRG coding quality. It was not fair for a hospital to be assumed 'capable' of producing good codes without qualified physicians and/or coders.

To our knowledge, this study is the first national survey to explore the structural and process components of coding practice, that might affect DRG coding quality. In terms of structure, we found that the use of software, number of medical statisticians, and experience of physicians seemed to be the most important. SCAD hospitals are more likely to have fewer medical statisticians, fewer certified coders, and less experienced physicians. Our previous case study revealed that, with inadequate numbers and inequitable distribution of certified coders, hospitals have tried to survive by using part-time coders from other disciplines, especially nurses [1]. This survey expanded the point further by suggesting that SCAD hospitals are more likely to face such problems than non-SCAD hospitals.

The current production of medical statisticians has been limited whereas the actual task is not necessarily about coding. In Thailand medical statistician is a job position that requires undergraduate-level training and usually is responsible for analyzing patient information [1]. Although the Thai DRG anticipated medical statisticians to be trained and certified to work as coder, a survey of 322 hospitals in 2001 revealed that only 59.87% of the hospital had medical statisticians worked as coders; but as many as 46% of them were considered 'part-time coders' as they had to be responsible for other jobs as well [23].

Based on the seven steps of the hospital coding process we reported earlier [1], the cross-function phenomenon also occurs with other health care professional disciplines as well. This study adds to the case study findings by quantifying the number of hospitals that allows such phenomenon to occur. For example, discharge summary has been assumed to be done by physicians and therefore used as a gold standard to see if the codes assigned by hospital coders are correct. However, nurses or medical statisticians are indeed the primarily responsible staff for discharge summary in some hospitals. Nurses have played important roles in almost all steps of the hospital coding process but they have not been formally recognized. While an experienced nurses can become certified coders, their contribution might not be counted toward job promotion in public hospitals.

We also are proposing development of a new tool called Hospital Coding Practice Scale, which can indirectly explore the DRG creep phenomenon. The audit by external peers has been a main mechanism to assess the quality of discharge summary as well as diagnosis and procedure codes but the results cannot be used to judge hospital intention to game the system. By using this measurement model, one can classify hospitals based on the domains they focused on and the profiles they fall into. We hypothesize that DRG creep is more likely among hospitals who focus mainly on reimbursement (profiles 3, 5, 6; Table 3). Further studies are required to ensure the validity, reliability, and feasibility of this tool.

The generalizability of the findings from this study is limited by low response rate. This was actually anticipated because some hospitals might be cautious to provide such detailed and confidential information about coding practices. The difference in both response rates and characteristics between SCAD and non-SCAD hospitals is another limitation of this study that does not allow a direct comparison of various aspects between the two groups. Also, we were unable to explore some other factors that might affect hospital coding practice. For example, various functionality of the software can affect the hospital coding practice.

## Conclusion

SCAD and Non-SCAD hospitals were different in many aspects, especially the number of medical statisticians, experience of medical statisticians and physicians, as well as number of certified coders. The findings suggested that hospital providers should not be assumed capable of producing high quality DRG codes, especially in resource-limited settings.

## Competing interests

The authors declare that they have no competing interests.

## Authors' contributions

KP conceived of and designed the study, carried out the survey, analyzed the data, and drafted the manuscript. DGW participated in its design, and helped to draft the manuscript. HR helped to revise the manuscript. CR helped to draft and revised the manuscript. All authors read and approved the final manuscript.

## Pre-publication history

The pre-publication history for this paper can be accessed here:

http://www.biomedcentral.com/1472-6963/11/290/prepub
